# Ischemic stroke and intestinal flora: an insight into brain–gut axis

**DOI:** 10.1186/s40001-022-00691-2

**Published:** 2022-05-25

**Authors:** Wenjie Hu, Xiangyi Kong, Hui Wang, Yunqing Li, Yimin Luo

**Affiliations:** 1 Department of Biological Science, Jining Medical University, Rizhao, Shandong China; 2 Institute of Neuroregeneration & Neurorehabilitation, Qingdao University, Qingdao, Shandong China; 3 Department of Pathogenic Biology, Jining Medical University, Jining, Shandong China

**Keywords:** Ischemic stroke, Intestinal flora, Brain–gut axis, Dysbiosis, Metabolites

## Abstract

Stroke is a type of cerebrovascular disease that significantly endangers human health and lowers quality of life. This understandably places a heavy burden on society and families. In recent years, intestinal flora has attracted increasing attention from scholars worldwide, and its association with ischemic stroke is becoming a hot topic of research amongst researchers in field of stroke. After suffering from a stroke, intestinal microbial dysbiosis leads to increased intestinal permeability and activation of the intestinal immune system, which in turn leads to ectopic intestinal bacteria and pro-inflammatory cells that enter brain tissue through the damaged blood-brain barrier. This exacerbates ischemia-reperfusion injury. Interestingly, after a stroke, some metabolites produced by the intestinal flora attenuate ischemia-reperfusion injury by suppressing the post-stroke inflammatory response and promotes the repair of neurological function. Here we elucidate the changes in gut flora after occurrence of a stroke and highlight the immunomodulatory processes of the post-stroke gut flora.

## Background

Stroke is an acute cerebrovascular disease, which can be caused by either sudden rupture of cerebral vessels or vascular occlusion; this is also referred to as hemorrhagic stroke (HS) and ischemic stroke (IS), respectively [1]. The incidence of ischemic stroke is significantly higher than that of hemorrhagic stroke, accounting for roughly 80% of the total incidence of cerebrovascular injury. The interruption of blood supply to brain, accompanied by hypoxia, further cause IS related nerve damage. Ischemic stroke was caused by a variety of risk factors and brought a heavy burden upon the patients’ family as well as society in general [2]. The most important risk factors are hypertension, diabetes and atherosclerosis. Ischemic stroke is also a complex disease caused by a variety of environmental and genetic factors. Long-term domestic and foreign studies have shown that the risk factors of IS are made up of two categories, namely, non-modifying risk factors [3] (gender, age, genetic factors, family history and race.) as well as modifying risk factors [4] (hypertension, abnormal blood glucose, hyperlipidemia, atrial fibrillation, high homocysteine, and bad living habits.) Intervention refers to the ability of controlling the risk factors, especially the most dangerous, which are hypertension and diabetes, to reduce the incidence and mortality of this disease.

Intestinal flora refers to all microorganisms in the human gastrointestinal tract, comprising of between 15,000 ~ 36,000 bacterial species, which represents mostly the Firmicutes and Bacteriodetes phyla [5–7]. Beside bacteria, Archaea and eukaryotes, viruses as well as bacteriophages are also included in intestinal flora [8]. Intestinal flora has the ability to regulate the metabolic activity of the host, as well as regulate the intestinal immune and biological barriers [9]; thus, it has a role of maintaining the health state of the host [10]. The total number of bacteria and species that makes up the intestinal flora can be affected by many factors, such as environment [11], diet [12], medications and genetics. Intestinal flora and its surrounding intestinal environment are collectively referred to as the intestinal micro-ecosystems, which functions to maintain the homeostasis of the internal environment under normal conditions for humans and animals. Once the intestinal micro-ecosystems lose its homeostasis, various diseases occur, which may also involve the central nervous system [13–15]. Intestinal microecosystem disorders can change the microenvironment of the intestine, affect the function of intestinal absorption and metabolism, subsequently affecting the risk factors of IS [16] directly or indirectly. In addition, the enteric nervous system, known as the human “second brain”, can interact with the central nervous system, autonomic nervous system [17], hypothalamus-pituitary-adrenal axis and other structures to form a two-way regulatory axis, the brain-gut axis. Intestinal flora can also decompose fermented food ingredients and produce a series of metabolites [18–20] that play an important role in the brain-gut axis. It can form a network of nerve, immune and endocrine regulation by stimulating neuroendocrine and conduction pathways, which is the “flora-gut-brain” axis. Changes in intestinal flora can alter the intestinal defence function and intestinal permeability [21], which affects both the enteric nervous system and central nervous system.

At the same time, intestinal flora plays an important role in the development of central nervous system [22]. Studies have shown that gut microbiota can regulate a series of neurotrophic factors or proteins that are involved in brain development and plasticity, such as brain-derived neurotrophic factors [23], synaptophysin and postsynaptic dense region proteins. The sterile state of sterile animals can lead to changes in the nervous system, such as increased permeability of blood-brain barrier (BBB). Microglial cells are different from traditional bacterial colonization animals in morphology and function [24]. In addition, intestinal flora is also involved in the regulation of central nervous system activities, such as anxiety, depression and stress response [25]. Normal and steady intestinal flora plays a very important role in maintaining normal brain function and repair. When the balance of the intestinal flora changes, it can increase the risk of stroke through different mechanisms.

## Intestinal flora and its products cause stroke by inducing atherosclerosis

**Fig. 1:**
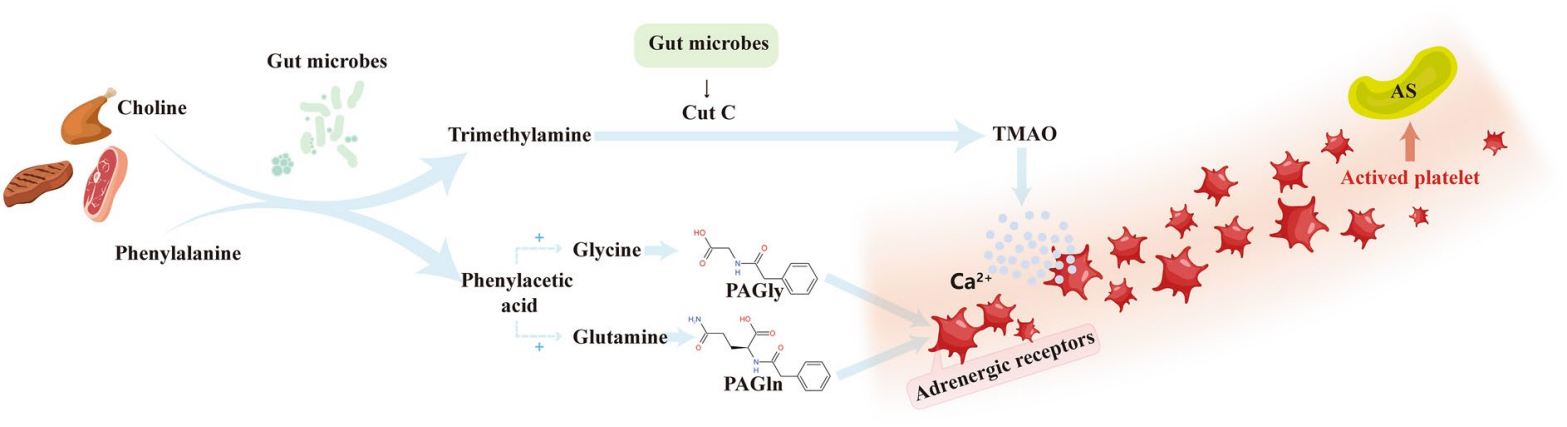
Some intestinal metabolites promote the development of atherosclerosis. Choline in food is transformed into trimethylamine by the action of intestinal bacteria, and the latter is formed into TMAO by the action of a specific group of bacteria containing the CutC gene. TMAO evokes the release of intracellular calcium stores and promotes platelet activation and atherosclerotic plaque formation. Phenylalanine in food is converted to phenylacetic acid by the action of porA gene-containing enteric flora, which synthesizes PAGln or PAGly with glutamine or glycine and binds to platelet adrenergic receptors to induce platelet hyperreactivity and promote atherogenic plaque formation

Platelet activation, aggregation and atherosclerotic plaque formation are important pathogeneses of ischemic stroke (Fig. 1). Recent studies have shown that intestinal flora play an important role in the occurrence of atherosclerotic plaques. Intestinal flora can affect the occurrence of atherosclerosis in three different ways: (1) Bacterial infections activates the immune system [26] by influencing various immune cells [27]. Moreover, TLR expression by macrophages further leads to the increase of proinflammatory cytokines and chemokines, which accelerates the progression of atherosclerotic plaques and leads to the formation of vulnerable plaque. Microbes that have been shown to promote atherosclerosis include Porphyromonas gingivalis [28], Aggregatibacter actinomycetemcomitans, Chlamydia pneumoniae [29, 30] in addition to others. (2) Intestinal flora metabolism of food such as cholesterol and fat affect the formation of atherosclerotic plaque [31]. Transplantation of pro-inflammatory microorganisms can reduce the types of microorganisms that produce short-chain fatty acids (SCFA) in mice, enhance the inflammatory response and promote the formation of atherosclerosis [32]. Certain kinds of bacteria such as L. rhamnosus GG (LGG) or pharmaceuticals telmisartan (TLM) supplements can alter bacterial genera and reduce α-diversity, which has significant correlations to atherosclerotic plaque size, plasma A-FABP and cholesterol level [33]. (3) Certain metabolites such as trimethylamine N-oxide (TMAO), which is produced by intestinal flora, promotes atherosclerotic plaque formation by activating platelet activity. The TMAO pathway is considered to be the most direct pathway, where intestinal flora influences the process of atherosclerosis [34, 35].

Choline from the diet is metabolized by intestinal microorganisms to produce trimethylamine, which is oxidized to TMAO after entering the liver via liver-gut circulation. TMAO promotes the release of intracellular calcium ions extracellularly in a platelet activator-dependent manner, which thereby mediates the high reactivity of platelets and increases the risk of thrombosis [36, 37].

In addition to animal experiments, clinical studies have also shown that TMAO is involved in the occurrence of atherosclerosis, which is significantly associated with the risk of cardiovascular and cerebrovascular events. In a study conducted by Tang et al. [38] which involved 4007 subjects who were followed up for 3 years to study the relationship between the concentration of plasma TMAO and the risk of cardiovascular/cerebrovascular events. The results showed that TMAO was positively correlated with the risk of thrombosis in a dose-dependent manner, and this effect was independent of traditional cardiovascular and cerebrovascular disease risk factors. Yet in the study of Yin et al. [39] did not find elevated plasma TMAO levels in stroke patients or transient ischemic attack (TIA) patients. Their study also analyzed the differences in intestinal flora composition and TMAO levels in asymptomatic patients with atherosclerosis, stroke and TIA. The results showed that the levels of TMAO and the composition of intestinal flora were similar in asymptomatic atherosclerosis patients, with or without carotid plaques. However, the composition of intestinal flora in patients with stroke or TIA was significantly different from that of patients with asymptomatic atherosclerosis. Notably, even though the TMAO level was not as high as expected, the level was still lower than that observed in patients with asymptomatic atherosclerosis. Tang et al. [38] explained that the medications used to treat stroke may reduce the level of TMAO. Therefore, the correlation between intestinal flora product, TMAO, and ischemic stroke needs further confirmatory research. The microbial cut C gene was found to mediate the TMA/TMAO conversion, as well as increase infarction size; thus this gene can be thought to promote impaired neurological function by genetically engineering modified bacterial transplants in germ-free mice. In other words, gut microbes can exacerbate infarcts by producing TMAO [40].

In addition to TMAO, other intestinal flora metabolites that can activate platelets include PAGln and PAGly [41]. They represent phenylacetic acid, which is consumed from the diet, subsequently converted into phenylalanine by intestinal flora and ultimately into glutamine and glycine, respectively. PAGln and PAGly are similar in structure to adrenergic receptors and can, therefore, bind to platelet β2 receptors in the body, able to activate platelets to promote thrombosis. However, some studies have found that PAGly can activate the Gαi/PI3K/AKT signal cascade by stimulating β2AR, thereby inhibiting cell apoptosis and reducing the area of myocardial infarction caused by I/R injury. However, high-dose treatment will cause a higher mortality rate [42]. It can be observed that the role of PAGly in the body is closely related to its dose. However, the role of PAGly after ischemic stroke, and the mechanism by which it functions, have not yet been reported, and needs further exploration (Fig. 1).

It is worth mentioning that Porphyromonas gingivalis, located in the oral cavity, is also found to be associated with the development of stroke [43, 44].

## Changes in intestinal flora can affect brain repair after stroke

The “flora-intestine-brain” axis is a new concept. It is a prerequisite hypothesis, which showed that in the model of middle cerebral artery occlusion (MCAO), intestinal flora has a significant impact on stroke prognosis. The study of Benakis et al. [45] declared that flora imbalance, caused by antibiotics, could reduce the α-diversity of intestinal flora and improve prognosis; the histology showed a decrease in the volume of ischemic tissue. This effect is mainly due to the decrease of IL-17^+^γδT cells and the increase of Treg cells in the small intestine, thereby limiting the infiltration of harmful substances into the brain membrane of IL-17^+^γδ T cells. Sun et al. [46] found that butyric acid bacteria can reduce cerebral I/R injury in diabetic mice by regulating intestinal flora. 16S rRNA gene sequencing, combined with LC–MS analysis, showed that in rats with IS, the intestinal flora and plasma metabolites changed. Moreover, it showed that the abundance of Proteobacteria Firmicutes and Deferribacteres was significantly different between Sham and IS groups. The gut microbiota was strongly correlated with the dysregulated metabolites [47]. Xu et al. [48] found that MCAO mice extended rapid and dynamic dysbiosis. The increase of Enterobacteriaceae bacteria aggravates cerebral infarction by enhancing systemic inflammation. Related studies have shown that dysregulation of microflora is one of the reasons for poor prognosis of patients with primary stroke. The use of aminoguanidine or superoxide dismutase to reduce nitrate production, or by using tungstate to inhibit nitrate respiration, can inhibit the overgrowth of Enterobacteriaceae bacteria, reduce systemic inflammation and reduce risk of cerebral infarction. These therapeutic effects are dependent on the gut microbiota, which indicates the translational value of the brain-gut axis in the treatment of stroke. Wang et al. [49] proved that in patients with T2D, after AIS, the serum levels of lipopolysaccharide (LPS) and D-lactate (DLA) clearly increased; moreover, she showed that butyrate-producing bacteria including Lachnospira, Blautia, and Butyricicoccus decreased. After BS was replenished the mice showed lower levels of proinflammatory cytokine and exhibited a significantly smaller infarction volume. It also showed that fecal transplantation could attenuate ischemic stroke injury by protecting the BBB. MCAO models of pigs [50], after 1 day of stroke, also showed a reduction in the microbial diversity, and on the third day the lesion volume was negatively correlated with microbial diversity. In relation to the models, the abundance of Proteobacteria was significantly increased, while Firmicutes and lactic acid bacteria, Lactobacillus, decreased on the third day poststroke. The aforementioned results (from a pig model) suggest the plasticity of the gut microbiome during the acute period of stroke and its influence on brain damage.

## Changes in intestinal mucosal permeability affect stroke outcome

An intact intestinal mucosal barrier is an important defensive line for the body to protect against adverse external factors. When acute ischemic stroke occurs, the intestinal mucosal permeability is altered for various reasons, generally manifesting as increased permeability. This results in a large number of toxic products that enter the blood circulation through the intestinal mucosa, which then enter the nervous system causing damage. The impairment of intestinal barrier function in patients with cerebral infarction may be related to the following factors, outlined in the next three paragraphs.

### Ischemic stroke leads to reduced expression of intestinal junction proteins

The intestinal mucosa, including the structure of epithelial tight junctions (TJs), are composed of multiple protein subunits [51], of which Claudins and occludins are particularly important because of their key structural roles. Many studies [52, 53] have examined their expression levels as a marker of altered mucosal permeability. There is a reduced expression of zonula occludens-1 (ZO-1), occludin, and claudin-1 after stroke [54]. Cerebral infarction decreases the expression of intestinal mucosa tight junction proteins, Occludin, which leads to the destruction of tight junctions, damages the intestinal barrier, and increases intestinal permeability. Xia et al. [55] found that compared with the Sham group, the expression of ZO-1, VE-cadherin, Occludin and Claudin-5 in the rats from the MCAO group appeared to be reduced in different degrees. Shengui Sansheng Pulvis (SSP) administration restored the expression of these proteins in the intestinal mucosal epithelium while reducing MCAO-induced brain edema, and increased VIPR1/2 expression in the OGD blood–brain barrier models, reducing endothelial injury.

### Increased intestinal epithelial permeability induced by microRNA after stroke

MicroRNA is a kind of small non-coding ribonucleic acid that participates in various pathophysiological processes of the body. MiR-21-5p is one type of miRNAs. Wu et al. [56] found that miR-21-5p was significantly increased in the serum of patients with cerebral infarction. Studies have found that miR-21-5p can increase intestinal epithelial permeability by up-regulating small GTPase-ADP-ribosylation factor 4 (ARF4) [57]. The ability of miR-21-5p to increase vascular permeability has been similarly demonstrated in studies of colorectal cancer and may be related to its targeting of Krev interaction trap protein 1 (KRIT1) and activation of the β-catenin signaling pathway.

### Dysregulated intestinal flora, after stroke, produces toxic metabolites acting on the intestinal mucosal epithelium

**Fig. 2:**
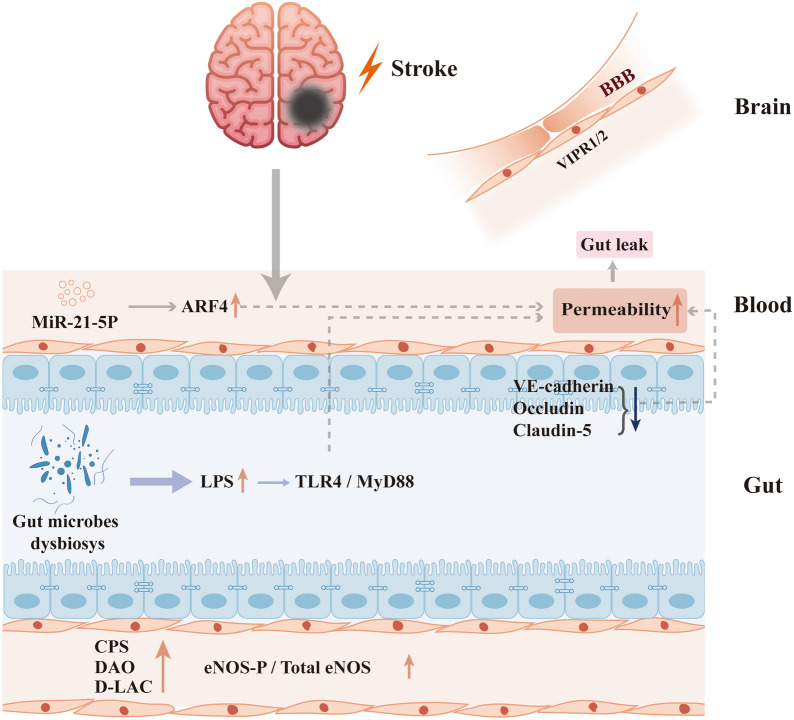
Post-stroke intestinal changes and their impacts on cerebral organization. Stroke causes a reduction in the expression of intestinal epithelial tight junction proteins including VE-cadherin, Occludin and Claudin-5; more LPS is produced by post-stroke intestinal flora, which induces damage by binding to TLR4/MyD88 in the downstream inflammatory response; LPS also contributes to an increase in eNOS-P/total eNOS, causing vascular endothelial damage; stroke causes an increase in miR-21-5p and further upregulated ARF4; the aforementioned factors combined lead to increased intestinal mucosal permeability and leaky gut. The blood LPS, DAO and D-LAC elevated after vascular endothelial injury and BBB endothelial injury accompanied by VIPR1/2 decreasing

Kurita et al. [58] detected LPS and K99 pili protein localization in the brain 24 h after stroke, existing in the Iba-1 positive microglia, neurons as well as endothelial cells. The result indicated that ischemia-induced Enterobacteriaceae proliferation led to increasing luminal LPS concentration, weakened the tight junction of epithelial cells and promoted LPS circulatory system entry. Singh et al. [59] found that stroke could affect the composition of intestinal flora. When intestinal flora is imbalanced, opportunistic pathogens can produce a variety of harmful substances, such as lipopolysaccharide. Lipopolysaccharide is the cell wall component of Gram-negative bacteria, also known as endotoxin, which can affect the tight junction of intestinal epithelium and increase intestinal permeability by mediating the Toll-like receptor (TLR)4/MyD88 signal transduction pathway. TLR-4 positive cells started to increase in number 1 h after MCAO and continued until 22 h. Specific knockdown of TLR-4 was able to produce a protective effect against ischemic stroke. It is evident that TLR-4 is an important target in stroke [60]. Gut microbiota disruption could cause cerebral endothelial dysfunction through eNOS activity decrease [61]. Stroke can lead to increased abundance of Gram-negative Enterobacteriaceae bacteria and further increased circulatory LPS levels [58, 62], which can trigger inflammation via TLR-4 [63] and alter intestinal mucosal ligand protein expression levels leading to a leaky gut. Meanwhile, LPS induces an inflammatory response, which further aggravates stroke injury. This suggests that stroke and altered intestinal flora are biphasic. In the cerebral artery lysates of antibiotic-treated rats, the eNOS-P/total eNOS ratio was decreased compared to the control subjects. Using antibiotics cause the disruption of gut microbiota and as a result lead to cerebral endothelial dysfunction. However, this study is opposite to the study of Benakis et al. [45]. The intestinal barrier is one of the basic defence lines of the body against the external environment, which plays an important role in ensuring the stability of the body internal environment. Blood DAO (diamine oxidase), D-LAC (Dlactate) and endotoxins [64] are reliable indicators that reflect the function of the intestinal barrier. Mice with hyperuricemia were found to possess a damaged intestinal barrier as well as an enhanced intestinal permeability, which lead to an induced inflammatory process. Elevated serum uric acid levels were seen to be associated with an increased risk of acute ischemic stroke; however, the mechanism is not clear. Potentially, by combining the changes to the characteristics of intestinal permeability acute ischemic stroke and hyperuricemia, we could elucidate the correlation. In fact, Crapser et al. [65] had the similar results in animal studies. However, several studies [66] have concluded there is insufficient evidence for changes in the morphology and expression of permeability proteins in the intestinal mucosal epithelium after MCAO (Fig. 2).

## Cytokines released by gliacytes and other cell type, post ischemic stroke, can either aggravate or relieve brain damage

Ischemia and hypoxia in brain tissue from various causes trigger a series of cascade reactions, including glial cell activation and release of inflammatory mediators, leading to the activation of endothelial cells, which express adhesion molecules and recruit inflammatory and immune cells from the circulation to the site of stroke injury. The simultaneous release of DAMPs/cytokines as well as the activation of the vagus nerve results in intestinal motility disorders, intestinal disorders and increased intestinal permeability. Vila et al. [67] found that serum concentrations of IL-6 and TNF-α, at the time of admission, in stroke patients are strongly associated with early neurological deterioration. However, the specific sources of IL-6 and TNF-α were not mentioned in this study. Several studies [68–70] have shown that the period after an episode of a stroke can cause increased expression of pro-inflammatory inflammatory factors in the serum and within brain tissue. This exacerbates local or systemic inflammatory responses and further aggravates brain tissue damage. Primary astrocytes were seen to express only low levels of TLR2, TLR4, TLR5 and TLR9 under resting culture conditions, but their mRNA expression levels were significantly upregulated when cells were exposed to specific bacteria-derived ligands [71]. TREM1 is produced by Ly6C^+^MHCII^+^ macrophages in the lamina propria of the intestinal mucosa after a stroke; its ability to increase mucosal epithelial permeability promotes bacterial translocation across the intestinal barrier into brain tissue [72]. This reflects the fact that peripheral TREM1 induces enhanced pro-inflammatory responses to brain-derived and gut-derived immunogenic components. Inhibition of TREM1 is able to reduce brain damage through this specific innate immune pathway. Early activation of PMN in ischemic brain tissue may be caused by the rapid release of danger-associated molecular patterns (DAMPs) and eventually leads to the secretion of IL-1b [73–75]. This is a process that promotes the onset of inflammatory vesicle formation by activating immune cell surface receptors and further activating the NLRP3 pathway. If the infiltration of the penumbra PMN is removed after the onset of stroke, the initial brain damage does not have a significant impact on the behavioral performance of the animal [76].

It is not difficult to discover that an inflammatory state is critical to evoke the neurotoxic potential of the invader. Resting-state PMN showed no neurotoxic activity in brain slices without ischemic pre-injury, and only lipopolysaccharide-activated PMN exhibited this effect. A similar increase in TREM-1 expression can occur during intestinal ischemia–reperfusion, but the use of the inhibitor, LP17, delays death in experimental animals [77]. The intestinal tract is the main immune organ that is equipped with the largest immune cell pool, accounting for more than 70% of the whole immune system [78]. Displaced intestinal microorganisms can: (1) stimulate intestine-related lymphoid tissue, stimulate the differentiation of immune cell subsets; (2) promote the occurrence of inflammatory response; and (3) aggravate the possibility of systemic inflammatory response and multiple organ dysfunction.

Microglia are derived from the myeloid cells of the yolk sac, which are localized to the central nervous system early in individual development and are resident immune cells of the central nervous system [79]. The dendritic and axonal morphology of germfree mouse neurons is affected during development and such developmental defects are often associated with an immature microglial phenotype. This means gut microbial colonization during the development of the brain is crucial [80, 81]. Microglia are capable of proliferation and polarization and can change when in a pathological situation from a branching resting state to an amoeboid activated state [82–84].

In addition, T lymphocytes play an important role in the stroke process. The dysbiosis, induced by the acute phase of stroke, promotes pro-inflammatory Th1 and Th17-mediated immune responses derived from intestinal Peyer's lymph nodes and contributes to brain injury [59, 85]. When intestinal microecological homeostasis is achieved after FMT, the number of Treg increases within the ischemic brain region [86]. In chronic colitis, combined with stroke, intestine-derived CD4 + T cells migrate from the intestine to the meninges and may interact with meningeal macrophages, leading to non-intestine-derived CD4^+^T cell infiltration and M1 and M2 microglia/macrophage imbalance, exacerbating brain injury in ischemic stroke [87]. At the same time, it can also promote the migration of immune cells from the intestine to the injury site of the cerebral infarction, and aggravate local injury. This may provide an insight into the positive correlation between the degree of intestinal barrier dysfunction and the degree of neurological deficit in patients with cerebral infarction.

## Bacterial ectopic location after stroke leading to the occurrence of infections in other tissues and organs

Stroke can lead to ectopic bacterial infections. A neurocentral injury such as a stroke can lead to a disruption of the original balance between the CNS and the immune system, secondary immunodeficiency or immunosuppression. Ultimately this leads to the development of infection [88, 89]. The bacteria belonging to ectopic infections are almost always species pertaining to bacteria native to the intestinal flora that enter the blood circulation and invade other tissues after stroke. One reason for this is due to increased permeability of the intestinal mucosa, colonizing and causing infection. The study of Wen SW et al. [90] demonstrates that exacerbated dysfunction of the intestinal barrier in advanced age promotes translocation of gut-derived bacteria and contributes to the increased risk to post-stroke bacterial infection. Tascilar et al. [91] found that in the animal MCAO model, there is post-stroke intestinal mucosal barrier disruption and bacterial translocation, which includes lung, liver, spleen and mesenteric lymph nodes. The most common pathogen is coagulase-negative Staphylococcus aureus. The impaired intestinal barrier function creates favourable conditions for intestinal microbial translocation.

However, Oyama [66] suggested no significant differences in intestinal mucosal changes in animals during the acute phase of stroke; moreover, their lung bacterial colonization may be related to the inadvertent aspiration of intestinal flora into the trachea and subsequently into the lungs during the operation of gavage. In addition, pro-stroke stress is also associated with bacterial translocation from the colon into other tissues (e.g. mesenteric lymph nodes, liver, and the spleen.), increases the inflammatory phenotype of the intestinal mucosa (e.g. COX-2, iNOS.) and reduces the amount of local secretion of IgA [92]. This pro-stroke stress is linked to stroke outcome [93, 94]. Regardless, post-stroke infection is the most common complication, as well as being the most serious complication; its mechanisms need to be further explored.

## Changes of intestinal flora are closely related to post-stroke depression

The brain-gut axis is a two-way regulatory axis of the interaction between the brain and the gastrointestinal tract. Gastrointestinal discomfort is often accompanied by emotional reactions, which in turn can activate the neural activities of the related central nervous system parts. At the same time, the regulatory information is transmitted down to the gastrointestinal tract through the brain-gut axis, changing its dynamic and secretive functions, activating intestinal mucosal immunity and affecting the intestinal mucosal barrier function. For example, in patients with gastroesophageal reflux, there is a strong correlation between anxiety and depression as well as gastrointestinal symptoms, such as gastric mucosal erosion. Furthermore, psychological or antidepressant treatment is effective for some patients [95, 96]. In psychiatric patients, depression and generalized anxiety disorder are often accompanied by gastrointestinal discomfort [97], and many patients with generalized anxiety disorder are often first diagnosed with a gastro-enterologic issue [98]. Thus, brain-gut axis dysfunction may play a role in the development of mental illness. However, regarding the underlying mechanism, current research tends to point towards the involvement of the gut flora [99, 100]. Under pathological conditions, the permeability of the BBB changes [101], various inflammatory factors enter the central nervous system. The inflammatory signal is transmitted to the central nervous system, and glial cells are activated through the NF-κB pathway to promote the occurrence of depression [102, 103].

Post-stroke depression is very common in the post-stroke population [104]. Patients suffering from the post-stroke phase, combined with cognitive impairment and depression, tend to have dysbiosis of the intestinal flora. PSCCID patients, compared to non-PSCCID patients, exhibit increased abundance of Proteobacteria, including Gammaproteobacteria, Enterobacteriales and Enterobacteriaceae, and decreased abundance of several short-chain fatty acid producing bacteria [105].

The administration of LPS was found to mimic depression-like behaviour in experimental animals. A significant inflammatory response in the central nervous system was observed, suggesting inflammatory responses induced by bacterial products such as LPS can affect the central nervous system and promote the development of depression [106, 107]. Chronic mild stress causes elevated IL-1β, COX-2 and PGE2 in blood and decreased 15d-PGJ2 expression in brain tissue. The use of antibiotics can reduce inflammation by inhibiting the TLR signalling pathway, so this target can be studied for depression [108]. Blocking or inhibiting toll-like receptors involved in central nervous system inflammation, and depression-like behaviour, induced by chronic mild stress, can both lead to improvement of inflammation and animal behavior [109–111]. Depression and post-stroke depression have similar clinical manifestations. Current studies have concluded that the pathogeneses of both are similar. However, recent studies also have shown the association between depression and gut flora are not specific to post-stroke depression, advising that research in this area needs to be further investigated. Therefore, further animal experiments and clinical studies are needed to explore the effect of intestinal flora on post-stroke depression.

## Mechanisms that can exert a protective effect against stroke through intestinal flora

The intestinal flora is also capable of producing metabolites that facilitate stroke recovery, of which SCFA is one of the most widely and intensively studied molecule. SCFA in humans includes high levels of acetic, propionic, and butyrate [112], as well as low levels of formate, valerate and caproate [113]. SCFA is actively absorbed into the circulation via monocarboxylate transporters (MCTs) [114] and can cross the blood-brain barrier [115, 116]. Clinical studies have found that lower SCFA levels are strongly associated with stroke and stroke-associated pneumonia (SAP) [117]. Fecal transplantation or SCFA supplementation improve stroke prognosis, with butyric acid having the most significant effect, increasing the abundance of beneficial lactobacilli and reducing intestinal mucosal permeability [118]. The intestinal microbiota of young and older mice was examined separately. We identified a high concentration of SCFA, and its producing strains, in the stool of young mice. SCFA-producing bacteria (Bifidobacterium longum, Clostridium symbiosum, Faecalibacterium prausnitzii and Lactobacillus fermentum) transplantation resulted in increased intestinal mucosal integrity, increased SCFA in the blood and brain tissue, increased Treg in brain tissue, decreased IL-17^ +^ γδ T cells, reduced neuro-inflammation, and significantly improved behavioural scores [119]. Sadler et al. [120] reported a number of notable findings: (1) SCFA levels in the blood decreased after stroke; (2) artificial SCFA supplementation reduced the expression of CD68 in Iba-1^+^ microglia, as well as decreased the number of microglia activation, which reduced the inflammatory response in the brain group after stroke. This in turn was stipulated to enhance synaptic plasticity in the cortical semidark zone and improve stroke prognosis and cortical reconstruction. This suggests that SCFA, produced by intestinal flora, may serve as the basis of metabolites for the brain-gut axis to function. Not only complex short-chain fatty acids, but also SCFA species such as butyrate alone can exert neuroprotective effects [121–123].

Once bile is secreted in the intestine, bile acids are metabolized into a pool of bile acid by the action of intestinal flora. After metabolism, primary BAs such as CA, CDCA and UDCA are formed, and further secondary BAs including DCA and LCA are produced. These metabolites can bind to various receptors in the brain, such as FXR [124], TGR5 [125], NMDAR [126], and PXR [127], subsequently these molecules exert biological activities. TUDCA injection 1 h after ischemia increased intracerebral bile acid levels, reduced infarction size, and decreased neuronal apoptosis by increasing mitochondrial stability. This protective effect was maintained for at least 7 days [128]. TUDCA can reduce serum glutamate, TG, TC, and LDL-C levels, decrease inflammatory factor expression, increase SOD and GPX expression, reduce oxidative stress damage, and down-regulate the Nrf2 signalling pathway and apoptotic protein levels in cerebral ischemic rats, Thus, these effects exert neuroprotective effects [129]. Considering the wide variety of metabolites of bile acids and their ongoing discovery, the role of other species of bile acids in ischemic stroke needs further investigation. In addition, the neuroprotective effect of beneficial bile acid species can be induced by modulating the intestinal flora.

**Fig. 3:**
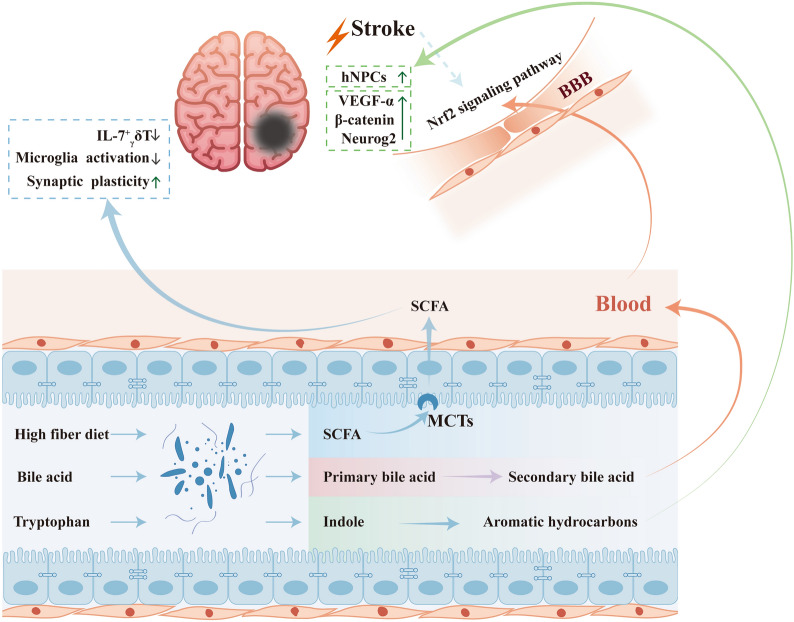
Certain intestinal flora metabolites promote post-stroke recovery. Certain foods, such as high-fiber foods, can be metabolized by intestinal flora to produce SCFA, which is transported and absorbed by MCTs and enters the brain, reducing IL-17 + γδ T cells, diminishing activated microglia, and increasing synaptic plasticity; bile acids are transformed by intestinal bacteria into primary bile acids, which are then transformed into secondary bile acids and enter the blood or cross the blood–brain barrier, bind to receptors and upregulate SOD and GPX Tryptophan in food can be metabolized by enterobacteria to indole, which binds to intestinal mucosal aromatic hydrocarbon receptors and promotes the growth rate of human neural progenitor cells (hNPCs) by promoting β-catenin, Neurog2, and VEGF-α expression

Tryptophanase expressing microorganisms in the intestine converts tryptophan to indole, which upon binding to aromatic hydrocarbon receptors promotes the expression of β-catenin, Neurog2, and VEGF-α and promotes neurogenesis in the hippocampus [130]. This is highly consistent with the findings of Möhle et al. [131], which found that antibiotic treatment reduced hippocampal neurogenesis and memory formation in adult mice; however, adoptive transfer of Ly6C(hi) monocytes rescued this injury. Physiological levels of SCFA can promote the growth rate of human neural progenitor cells (hNPCs), and induce increased mitosis [132]. Promoting neurogenesis or neural stem cell regeneration can facilitate neurological recovery after stroke, thus intestinal flora may further improve stroke outcomes by promoting neural stem cell regeneration (Fig. 3).

In addition to the aforementioned, factors such as age at onset of stroke and gender [16, 133–135] can also influence the outcome by affecting the gut flora. MCAO using SD rats of different genders revealed that male SD rats had a more pronounced increase in intestinal mucosal permeability, more elevated pro-inflammatory cytokines in the blood, and possessed a higher mortality and neurological deficits compared to female SD rats [136]. Compared to bacteria, the role of fungi [137, 138] in the gut has been poorly studied. In addition, the so-called intestinal dark matter, i.e. viruses [139, 140] (including phages), are also supposed to have a very high importance in the disease and less research has been done in this area. Hence factors that are able to alter gut flora need to be refined and subsequently integrated at an overall level in future studies. After all, each organism is inextricably linked to one another rather than independent of each other, and this is where the microbial-brain-gut axis is specifically presented. In short, the research on intestinal flora and ischemic stroke is still in its infancy. Intestinal flora is expected to become a new target for nerve protection through many pathways in post-stroke injury repair. It is believed that the treatment mode targeting intestinal flora in the future will play an important role in primary prevention and secondary prevention of ischemic stroke.

## Data Availability

These data were derived from the following resources available in the public domain: https://clarivate.com/webofsciencegroup/solutions/web-of-science/; https://pubmed.ncbi.nlm.nih.gov/.
